# The Origin of Plasma-Derived Bacterial Extracellular Vesicles in Healthy Individuals and Patients with Inflammatory Bowel Disease: A Pilot Study

**DOI:** 10.3390/genes12101636

**Published:** 2021-10-18

**Authors:** Emily Jones, Régis Stentz, Andrea Telatin, George M. Savva, Catherine Booth, David Baker, Steven Rudder, Stella C. Knight, Alistair Noble, Simon R. Carding

**Affiliations:** 1 Gut Microbes and Health Research Programme, Quadram Institute, Norwich Research Park, Norwich NR4 7UQ, UK; emily.jones@quadram.ac.uk (E.J.); regis.stentz@quadram.ac.uk (R.S.); andrea.telatin@quadram.ac.uk (A.T.); george.savva@quadram.ac.uk (G.M.S.); david.baker@quadram.ac.uk (D.B.); steven.rudder@quadram.ac.uk (S.R.); 2 Core Science Resources, Quadram Institute, Norwich Research Park, Norwich NR4 7UQ, UK; catherine.booth@quadram.ac.uk; 3 Antigen Presentation Research Group, Northwick Park & St. Mark’s Hospital Campus, Imperial College London, Harrow HA1 3UJ, UK; s.knight@imperial.ac.uk (S.C.K.); alistair.noble@pirbright.ac.uk (A.N.); 4 Norwich Medical School, University of East Anglia, Norwich NR4 7TJ, UK

**Keywords:** extracellular vesicles, gut bacteria, inflammatory bowel disease, dysbiosis, microbiota, plasma, 16S rRNA

## Abstract

The gastrointestinal tract harbors the gut microbiota, structural alterations of which (dysbiosis) are linked with an increase in gut permeability (“leaky gut”), enabling luminal antigens and bacterial products such as nanosized bacterial extracellular vesicles (BEVs) to access the circulatory system. Blood-derived BEVs contain various cargoes and may be useful biomarkers for diagnosis and monitoring of disease status and relapse in conditions such as inflammatory bowel disease (IBD). To progress this concept, we developed a rapid, cost-effective protocol to isolate BEV-associated DNA and used 16S rRNA gene sequencing to identify bacterial origins of the blood microbiome of healthy individuals and patients with Crohn’s disease and ulcerative colitis. The 16S rRNA gene sequencing successfully identified the origin of plasma-derived BEV DNA. The analysis showed that the blood microbiota richness, diversity, or composition in IBD, healthy control, and protocol control groups were not significantly distinct, highlighting the issue of ‘kit-ome’ contamination in low-biomass studies. Our pilot study provides the basis for undertaking larger studies to determine the potential use of blood microbiota profiling as a diagnostic aid in IBD.

## 1. Introduction

The human body harbors on average 3.8 × 10^13^ microbes, termed the microbiota [1], the largest community of which resides in the gastrointestinal tract (GIT). Comprising bacteria, viruses, fungi, protozoa, and archaea, the intestinal microbiota contributes to the life-long health of the host, playing key roles in digestion, the provision of essential amino acids and micronutrients, and in the maturation and maintenance of the host immune system as well as maintaining intestinal epithelial barrier integrity [2]. Associations have been demonstrated between an altered structural composition of the microbiota (dysbiosis) and many human diseases [3], in particular, those with a concomitant “leaky gut” and increased permeability of the intestinal barrier as seen in inflammatory bowel disease (IBD) [4]. IBD is a debilitating group of chronic diseases which causes relapsing–remitting inflammation of the GIT and affects millions of people worldwide [5]. IBD is primarily categorized into two diseases: Crohn’s disease (CD), which encompasses inflammation throughout the GI tract, or ulcerative colitis (UC), which affects primarily the colon. 

The intestinal microbiota of IBD patients characteristically shows reduced bacterial richness with a phylum-level decreased abundance of *Firmicutes* and associated increased abundance of *Proteobacteria* compared to individuals without IBD [6]. Whether this dysbiosis contributes to intestinal inflammation and if it causes increased intestinal barrier permeability is not known [7]. Increased permeability of the epithelial barrier enables luminal dietary antigens and microbial products, such as lipopolysaccharide (LPS) and other microbe associated molecular pattern (MAMP) molecules and nanosized bacterial extracellular vesicles (BEVs) expressing an array of MAMPs, to access the underlying lamina propria and vasculature [8]. The translocation of MAMPS into the blood stream can lead to increased systemic immune activation and the chronic gut inflammation that is characteristic of IBD [9]. 

BEVs are naturally produced by both Gram-negative and Gram-positive bacteria as a result of budding or blebbing of inner/outer cell membranes or explosive cell lysis [10,11]. As BEVs form, they encapsulate cytoplasmic and/or periplasmic proteins, enzymes, RNA, and DNA via incompletely understood selective sorting processes [12,13,14]. Some studies suggest that environmental factors and stressors encountered by the parental cells strongly influence the nature of BEV cargo [15]. The physiochemical properties of BEVs and their inherent resistance to physical, chemical, and biological insults make them ideal drug delivery vehicles in the hostile environment of the GIT, facilitating the delivery of their cargo to specific cells and locations within the host [16]. This role of BEVs was first elucidated for Gram-negative pathogens which use the BEVs they generate to deliver toxins and virulence factors to breach host defenses and allow parental cells to invade and establish infection within the host [8]. For commensal Gram-negative bacteria, the role BEVs play in microbe–microbe and microbe–host interactions is only just becoming clear. For example, we and others have shown that major human gut commensal *Bacteroides* spp., including *Bacteroides thetaiotaomicron,* generate BEVs expressing hydrolases and β-lactamases that can promote the growth or protect other gut bacteria from otherwise fatal doses of antibiotics, respectively [17,18]. They can also initiate host immune responses via their interaction with antigen presenting cells [19] and cross the intact intestinal epithelial barrier to reach distant tissues such as the liver and kidneys [20], suggesting BEVs play a key role as both short and long-distance delivery vehicles for bacterial effector molecules. 

A “leaky gut” is thought to precede the onset of disease symptoms, and patients with quiescent IBD and their family members often have subclinical intestinal inflammation [21]. Therefore, detection of a compromised gut barrier could provide an earlier diagnosis for patients and interventions that can halt the development of IBD symptoms. Current methods of IBD diagnosis require the presentation of intestinal inflammatory disease symptoms and often requires many tests for inflammatory biomarkers such as fecal calprotectin or C-reactive protein (CRP) alongside invasive investigations such as an endoscopy and biopsy samples [22]. However, these standard biomarkers can be insensitive, potentially reflecting inflammatory conditions of unknown origin. Therefore, comparing the presence of BEVs in the blood of both healthy individuals and those with diseases associated with a “leaky gut” holds the potential for clinical applications as novel biomarkers. 

Consequently, a number of studies using DNA (16S rRNA) sequencing have investigated the origin of the “blood microbiome”, indicating that the blood microbiome of healthy individuals comprises of DNA originating from mainly *Proteobacteria*, *Actinobacteria*, *Firmicutes,* and *Bacteroidetes* [23], although the source of this circulating DNA is often not described. BEV-associated DNA has been identified in patients with Alzheimer’s disease [24], Parkinson’s disease [25], dermatitis [26], asthma [27], cardiovascular disease [28], gastric cancer [29], biliary tract cancer [30], and COVID-19 [31]. However, few have investigated the origin of blood-derived BEVs in IBD. In a recent study aiming to define the biological properties of BEVs, Tulkens and colleagues described an increase in LPS-positive BEVs in the plasma of patients with intestinal barrier dysfunction, including those with IBD [32], independently of BEV-associated DNA. As the number of studies of BEVs in biological fluids such as blood increases, there is a growing need for universal, standard isolation methods, particularly for the isolation of BEV-associated DNA from low-biomass samples such as plasma. 

In this pilot study, we obtained a small number of plasma samples from healthy controls (HC) and a cohort of patients with an established diagnosis of Crohn’s disease (CD) and ulcerative colitis (UC) [33]. Our aim was to develop a fast, easy, and cost-effective method to isolate BEV-associated DNA from plasma to investigate the origin of the blood microbiota in IBD and HC samples. Samples were characterized by nanoparticle tracking analysis (NTA), protein analysis, and transmission electron microscopy (TEM) imaging. EV isolation methods were compared in an initial experiment prior to the main study. 

Using 16S rRNA gene sequencing, we found that there was no difference in the BEV-associated blood microbiota richness, diversity, or composition between IBD, HC, and protocol control groups, highlighting the issue of ‘kit-ome’ contamination in low-biomass studies.

## 2. Materials and Methods

### 2.1. Study Design

Archived plasma samples from a cohort of individuals enrolled in a study looking at microbiota immunity in IBD were used for this pilot study [33]. As previously described by Noble et al., patient samples were obtained from outpatient clinics at St Mark’s Hospital at Northwick Park Hospital, Harrow, UK, and included those with a diagnosis of Crohn’s disease (CD) or ulcerative colitis (UC). No patients were experiencing active, symptomatic disease at the time of sampling. Healthy control (HC) samples were obtained from individuals undergoing investigative endoscopy or from hospital staff and visitors. No HC individuals had a diagnosis of IBD. Plasma samples (4–5 mL) were obtained from a total of 23 individuals: 7 HC, 8 CD, and 8 UC (Table 1). All samples were stored at −80 °C prior to analysis. It should be noted that corresponding biomarkers for inflammation associated with IBD, such as fecal calprotectin, were not available for the samples used in this study.

A further two HC samples were obtained to use for initial protocol optimization before the main study whereby three EV isolation methods were compared to test 16S rRNA gene amplification and assess the efficiency of DNase I pre-treatment and extracellular protein removal.

**Figure 1:**
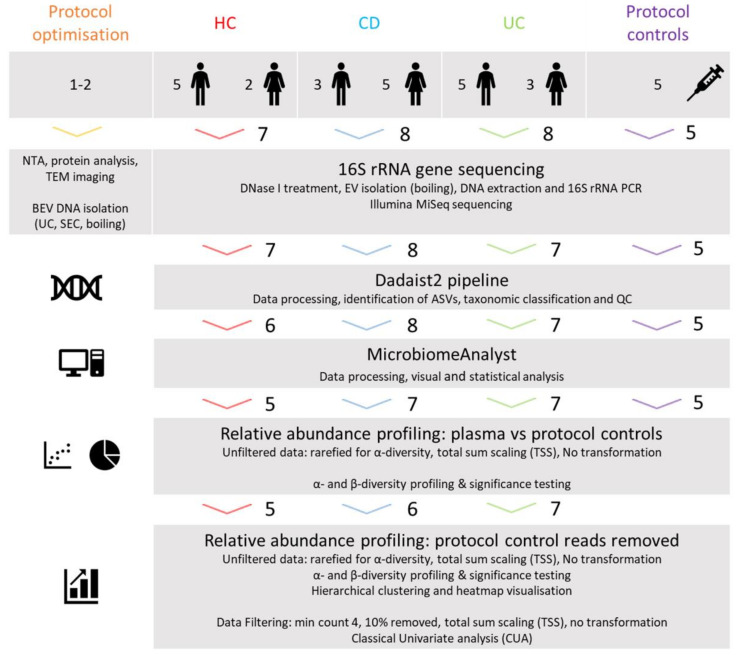
Study design overview. To characterize the BEV profile in HC and IBD plasma, archived samples from a cohort of healthy individuals (seven HC) and patients diagnosed with IBD (eight CD and UC) were utilized alongside five control samples representing each stage of the protocol. Two further HC samples were characterized and processed for protocol optimization. Samples were analyzed for 16S rRNA gene sequencing and data processed using the Dadaist2 pipeline. The processed data was analyzed using the MicrobiomeAnalyst web platform and evaluated using taxonomic profiling and significance testing. Healthy controls (HC), Crohn’s disease (CD), and ulcerative colitis (UC). The number of samples used at each stage of the study are indicated.

**Figure A1:**
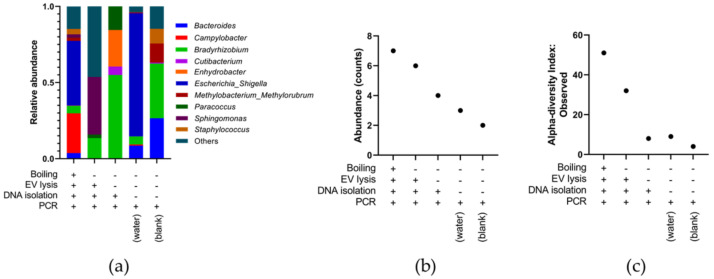
Taxonomic profile of the protocol control samples. (**a**) The relative abundance chart shows the top 10 most dominant taxa at the genus level. (**b**) Microbial abundance at the genera level (counts) of each protocol control sample reduces as each step of the protocol is removed. (**c**) Microbial richness measured by α-diversity also reduces as each step of the protocol is removed (ASV-level Observed Index: number of unique ASVs observed per sample).

For the main 16S rRNA gene sequencing study, five protocol controls were included for all stages of the protocol, including EV isolation, DNA extraction, and PCR to identify any contamination introduced, with one step of the protocol being removed for each sample: whole protocol, no boiling, no boiling or EV lysis, and PCR only (water or blank). Protocol control samples are outlined in Appendix Figure A1 and the study design is outlined in Figure 1.

### 2.2. Nanoparticle Tracking Analysis

Plasma nanoparticle tracking analysis (NTA) was performed using a ZetaView TWIN instrument according to manufacturer’s instructions (Particle Metrix GmbH, Inning am Ammersee, Germany). ZetaView software (version 8.05.12 SP1) was used to run a 2 cycle 11 position high frame rate analysis at 25 °C. Sample dilutions were carried out in ultra-pure water prior to analysis to fit within optimal detection range. Camera control settings: 80 sensitivity; 30 frame rate; 100 shutter. Post-acquisition parameters: 20 min brightness; 2000 max area; 5 min area; 30 trace length; 5 nm/class; 64 classes/decade.

### 2.3. Protein Concentration Measurement

Plasma protein concentrations were determined using a BCA Protein Assay Kit for low concentrations according to manufacturer’s instructions (Abcam, Cambridge, UK). Absorption was measured at 562 nm using a FLUOstar Plate Reader (BMG Labtech, Bucks, UK). All samples and standard controls were measured in duplicate.

### 2.4. Transmission Electron Microscopy

Plasma samples were visualized using negative staining with TEM. Briefly, 5 µL plasma diluted 1:10 in PBS were adsorbed to carbon-formvar coated copper EM grids (Agar Scientific, Essex, UK) for 30 s (un-processed) or 1 min (processed using SEC or boiling) before wicking off with filter paper and negatively staining with 2% Sodium (K) phosphotungstate solution (processed using SEC or boiling) or 0.5% Uranyl Acetate (unprocessed) for 1 or 2 min respectively. Grids were air dried before analysis using a FEI Talos F200C electron microscope at 17,500–57,000× magnification with a Gatan OneView digital camera. Images were processed using FIJI/ImageJ (v1.53c).

### 2.5. Extracellular Vesicle Isolation from Plasma

Plasma samples were thawed and centrifuged at 1500× *g* for 10 min at RT to remove cells and large particles and filtered through a 0.22 µm syringe filter prior to treatment with DNase I using a TURBO DNA-free Kit (Invitrogen, Loughborough, UK). EV isolation was then optimized using one of the following methods:

#### 2.5.1. Ultracentrifugation

Plasma samples (9 mL) were ultracentrifuged at 150,000× *g* for 2 h at 4 °C in a Ti70 rotor (Beckman Instruments, High Wycombe, UK) to pellet the EVs. The supernatant was discarded and the pellet resuspended in sterile PBS for further analysis.

#### 2.5.2. Size Exclusion Chromatography

**Figure A2:**
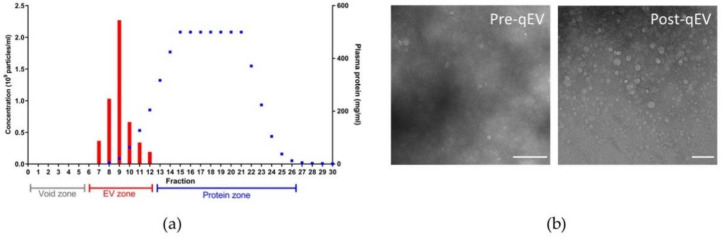
Plasma size exclusion chromatography (SEC) fractionation using qEV (IZON) columns. (**a**) Example fractionation profile from a qEV/35 nm SEC column loaded with 1 mL plasma. Concentration of particles (red bars) was measured using nanoparticle tracking analysis (NTA) with a ZetaView twin instrument (Particle Metrix). Total protein was measured using a BCA Protein Assay Kit for low concentrations (Abcam, blue points). The first 3 mL are void containing no particles (grey, void zone), plasma EVs typically elute in the next 3 mL (EV zone, red), and proteins in the remaining 7 mL (protein zone, blue). Sample *n* = 1. (**b**) Example of a plasma sample imaged using transmission electron microscopy (TEM) pre- and post-processing using a qEV/35 nm SEC column. Post-processing, extracellular proteins (white haze) are removed and intact, spherical vesicles can be visualized. Scale bar (200 nm).

Plasma samples (1 mL) were loaded onto a qEV/35 nm series size exclusion chromatography (SEC) column and fractions obtained according to manufacturer’s instructions (IZON Science, Lyon, France). Plasma BEVs typically elute in the 3 mL following the void volume and proteins in later fractions, so fractions 1–6 were pooled for further analysis. An example of HC plasma qEV SEC fractionation profile is provided in Appendix Figure A2a.

#### 2.5.3. Boiling

Plasma samples (1 mL) were boiled at 100 °C for 40 min in a thermomixer at 600 rpm (Eppendorf) followed by centrifugation at 13,000× *g* for 30 min at 4 °C to remove extracellular proteins. 

For optimization of the EV isolation protocol, all three methods (UC, SEC, and boiling) were compared. Following this, boiling was identified as the optimal method and all further samples for the main 16S rRNA gene sequencing study were processed for EV isolation by boiling only. 

### 2.6. DNA Extraction and Quantification

Samples were concentrated using Amicon Ultra 0.5 centrifugal filters with regenerated cellulose membranes and 10 kDa pore size (Millipore Limited, Feltham, UK) prior to lysis using an optimal two-step process. They were first incubated with a 10% SDS lysis buffer for 40 min at 37 °C followed by a second lysis step (lysis solution: guanidine hydrochloride) and DNA isolation using the GeneJET Genomic DNA Purification Kit according to manufacturer’s instructions (Thermo Fisher Scientific, Loughborough, UK). Total genomic DNA was collected in a final 100 µL volume. DNA was quantified using a Qubit 1× dsDNA High Sensitivity Kit (Thermo Fisher Scientific, Loughborough, UK). 

### 2.7. PCR and Gel Densitometry

To assess the efficiency of DNase I treatment to remove free DNA, plasma samples were spiked with *Bacillus subtilis* (ATCC 6633) DNA, and PCR used to assess DNA removal (before BEV lysis) using the strain specific primer pair: Bs_Fwd ACCATTGCGGTAGGTGCG and Bs_Rev GCGTTTGTCCAAGTCGGG [34]. For taxonomic profiling 16S, universal primers targeted the V3–V4 region of the 16S rRNA gene [35] and included the overhang Illumina index and sequencing adaptors: Fwd: 5′ TCGTCGGCAGCGTCAGATGTGTATAAGAGACAG and Rev: 5′ GTCTCGTGGGCTCGGAGATGTGTATAAGAGACAG. V3–V4 locus-specific 16S_Fwd TCGTCGGCAGCGTCAGATGTGTATAAGAGACAGCCTACGGGNGGCWGCAG, and V3–V4 locus-specific 16S_Rev GTCTCGTGGGCTCGGAGATGTGTATAAGAGACAGGACTACHVGGGTATCTAATCC were used. 

PCR products were separated by gel-electrophoresis and visualized under UV light using Midori Green Direct (Geneflow Limited, Lichfield, UK). In all PCR assays, a mock DNA extraction (water) and negative PCR control were included. 

### 2.8. 16S rRNA Gene Sequencing

Library preparation was performed using a two-step PCR amplification, first using Q5 High-Fidelity DNA Polymerase (New England Biolabs Limited, Hitchin, UK) and 16S universal primers targeting the V3–V4 region of the bacterial 16S rRNA gene as outlined above. The resulting single amplicon target of approximately ~460 bp was amplified using a second limited cycle PCR adding Illumina sequencing adaptors and dual-index barcodes. Samples were sequenced by Illumina MiSeq using paired 300 bp reads and MiSeq v3 reagents. 

### 2.9. Data Analysis

The 16S rRNA gene sequencing data was analyzed using the Dadaist2 v.1.0 pipeline [36], which pre-processes the reads using Cutadapt 3.3 [37] to remove the locus specific primers, fastp 1.2 [38] to produce SeqFu 1.4 [39] to identify the qualified regions for the identification of the amplicon sequence variants (ASVs) using DADA2 [34]. The taxonomic classification was performed within Dadaist2 using DECIPHER [40] against the SILVA database, release 138 [41].

Downstream analysis was performed using the MicrobiomeAnalyst web platform for data processing, statistical analysis, and visual analysis (updated version 16 February 2021) [42]. Firstly, a data integrity check was performed and samples with a low total read count (<100) were removed. Where the original data was used (relative abundance profiling, diversity profiling, and significance testing as well as hierarchical clustering and heatmap visualization), unfiltered data was scaled using total sum scaling (TSS). For α-diversity profiling, data rarefying was performed to even sequencing depth based on the samples with the lowest read counts. Where further filtering was performed to remove low abundance reads for classical univariate analysis (CUA), a cut-off of >4 counts in >20% of samples was used, and to remove low variance reads the cut-off was >10%. Visual data analysis was performed using stacked bar plots and heatmap clustering.

### 2.10. Statistics

All data are presented as the mean ± standard deviation (SD) with the indicated sample sizes. Using GraphPad Prism (v9.0.0), *p*-values for the relative taxa abundance were calculated using ordinary one-way analysis of variance (ANOVA) followed by Dunnett’s multiple comparisons test and Benjamini, Krieger and Yehotieli false discovery rate (FDR) test. Using MicrobiomeAnalyst, α-diversity was measured using the Observed Index (number of unique ASVs observed per sample) and one-way ANOVA to test differences between groups. β-diversity was measured using Bray–Curtis dissimilarity index and visualized using principal-coordinate analysis (PCoA) and differences between groups tested using permutational multivariate analysis of variance (PERMANOVA). Taxon-wise statistical comparisons of relative abundances were performed using CUA with either a *t*-test (two groups) or one-way ANOVA (>2 groups) to test differences between groups. The false discovery rate (FDR) was set to 0.05. Statistically significant features were considered when the corrected *p*-value < 0.05.

## 3. Results

### 3.1. Patient Characteristics

Participant demographics are shown in Table 1. Their ages ranged from 24 to 73 with a mean age of healthy controls of 43.9 ± 11.0, 49.0 ± 16.2 for CD, and 56.0 ± 9.46 for UC. Mean age of patients at IBD diagnosis was 36.6 ± 19.1 for CD and 39.9 ± 10.0 for UC. There were no substantial differences between symptoms and medications between IBD groups. 

### 3.2. Features of Plasma Nanoparticles 

**Figure 2:**
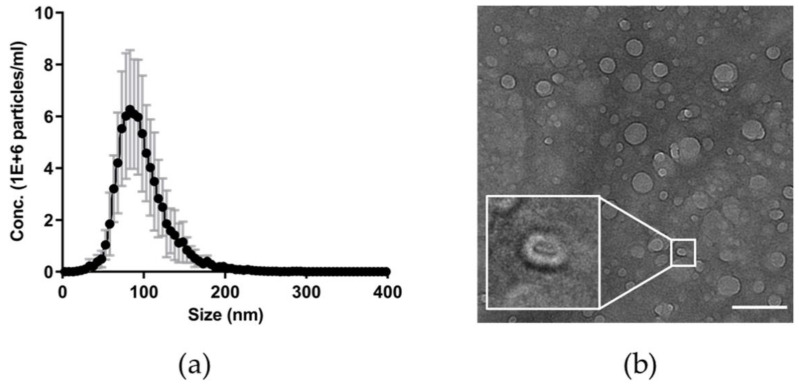
Characteristic features of plasma nanoparticles. Nanoparticle tracking analysis (NTA) was performed using a ZetaView twin instrument. (**a**) The average concentration of particles in plasma is 2.8 × 10^12^/mL with a heterologous hydrodynamic size range of 20–400 nm and a mean particle size of 94 nm in water. HC samples, *n* = 7. Points (black) represent the mean and the error bars (grey) represent the standard deviation (SD). (**b**) Transmission electron microscopy (TEM) identified intact, spherical vesicles in patient plasma with a heterogeneous size. Some of these particles displayed the classic cup-shape morphology typical of negatively stained BEVs (inset figure). Scale bar 500 nm.

The concentration and size distribution of plasma-derived nanoparticles was determined using a ZetaView twin instrument for NTA. The average concentration of particles in HC plasma was 2.8 × 10^12^/mL with a heterologous hydrodynamic size range of 20–400 nm in water (Figure 2a). 

Although TEM could not be performed on unprocessed plasma due to the presence of contaminating proteins (Appendix Figure A2b), imaging of qEV SEC processed samples confirmed the presence of intact, spherical vesicles in patient plasma (Figure 2b) with a heterogeneous size, with the majority displaying a bilayer morphology [43,44]. A small number of these particles were identified as having a classic cup-shaped morphology typical of negatively stained BEVs identified by TEM (Figure 2b inset).

The proportion of plasma EVs of bacterial origin is challenging to quantify by morphology from TEM imaging as they are generally indistinguishable from mammalian plasma EVs [45,46]. The majority of BEVs form by budding of the outer membrane (referred to as outer membrane vesicles (OMVs) [10]) and contain a single lipid bilayer. A small proportion forming outer-inner membrane vesicles (O-IMVs) containing a double lipid bilayer have also been described [47,48]; however, no O-IMVs were identified in our images. Further in-depth analysis is required for quantitative analysis using, for example, cryo-TEM [49,50] or immuno-EM using specific marker antigens for BEVs [32]. 

### 3.3. Optimising Bacterial Extracellular DNA Isolation from Plasma

To optimize the recovery of plasma-derived BEV DNA and remove contaminating extracellular proteins, three EV isolation methods were compared: ultracentrifugation, SEC (qEV columns), or boiling prior to processing all remaining samples with the chosen optimal method. For each method, 1–2 additional HC plasma samples were used alongside a protocol control sample (water). 

**Figure 3:**
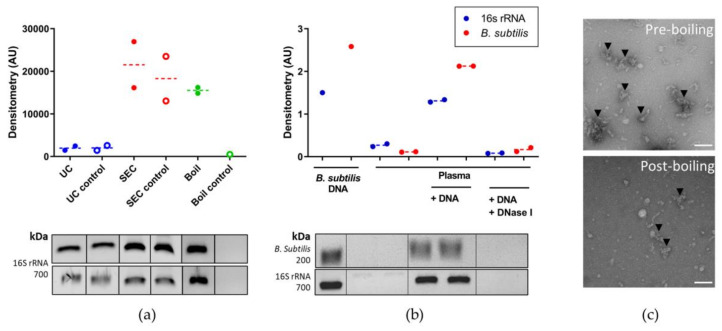
Optimisation of plasma-derived BEV DNA isolation. (**a**) EV isolation protocol comparison and densitometry of the corresponding PCR products per mL of plasma (AU). Ultracentrifugation (UC; blue), size exclusion chromatography using qEV columns (SEC; red) and boiling (boil; green). SEC and boiling methods show higher 16S rRNA gene amplification than ultracentrifugation. Only the boiling method identified significantly lower 16S rRNA gene amplification in the protocol control (water). The boiling method was identified as the optimal protocol for isolating BEVs for DNA extraction and was used for the main 16S rRNA gene sequencing analysis. Sample *n* = 1–2. The error bars represent the standard deviation (SD). (**b**) Extracellular DNA was eliminated from plasma samples by pre-treatment with DNase I. Chromosomal DNA from *B. subtilis* was used to spike plasma samples prior to DNase treatment to confirm the effectiveness of extracellular DNA removal (non-lysed samples). Plasma samples were processed for DNA extraction and amplified using primers specific for both *B. subtilis* (red) and the V3–V4 region of the 16S rRNA gene (blue). DNA was amplified by both primer sets (*B. subtilis* DNA), which was subsequently eliminated by DNase I treatment (plasma + DNA + DNase I) but not when DNase I was excluded from the reaction (plasma + DNA). Sample *n* = 1, analysed in duplicate except for *B. subtilis* DNA positive control. The error bars represent the standard deviation (SD). A faint 16S rRNA band corresponding to extracellular DNA was detected in the plasma control sample (*) which was eliminated by DNase I pre-treatment. (**c**) TEM imaging confirmed the reduction in extracellular protein aggregates (black arrowheads) after boiling. Scale bar 200 nm.

Following the EV isolation step, samples were further concentrated using centrifugal filters with regenerated cellulose membranes and a pore size of 10 kDa that have been shown to be the most efficient for EV recovery; both from eukaryotic (mammalian) and prokaryotic origin [51]. DNA was then isolated using the GeneJET Genomic DNA Purification Kit and after 16S rRNA gene amplification, densitometry of the corresponding PCR products was compared per mL of plasma. The SEC and boiling methods both showed higher 16S rRNA amplification than ultracentrifugation (Figure 3a). However, only the boiling method identified significantly lower 16S rRNA amplification in the protocol control (water, Figure 3a). This suggests the qEV SEC columns introduce bacterial DNA contamination and are not suitable for downstream PCR amplification applications. TEM imaging confirmed the reduction in extracellular protein aggregates (black arrowheads, Figure 3c) after boiling. Taken together, these results identified the boiling method as the optimal protocol for isolating BEVs for DNA extraction and this method was used for the main 16S rRNA gene sequencing analysis.

### 3.4. Identifying the Origin of Plasma Derived Bacterial Extracellular Vesicles by 16S rRNA Gene Sequencing

Extracellular DNA was eliminated from plasma samples by pre-treatment with DNase I. Chromosomal DNA from *B. subtilis* was used to spike one additional HC plasma sample prior to DNase treatment to confirm the effectiveness of extracellular DNA removal. Samples were then processed for DNA extraction and amplified using primers specific for both *B. subtilis* and the V3–V4 region of the 16S rRNA gene. Figure 3b shows that DNA was amplified by both primer sets, which was subsequently eliminated by DNase I treatment but not when DNase I was excluded from the reaction. A faint 16S rRNA band corresponding to free extracellular DNA was detected in the plasma control sample which was undetectable after DNase I pre-treatment (Figure 3b).

Due to the low-biomass nature of BEVs in plasma, low concentrations of DNA were obtained from all the main study samples. The mean concentration of DNA extracted from IBD plasma (CD 2.915 ng/mL ± 1.638 and UC 3.067 ng/mL ± 1.994) was comparable to HC (2.790 ng/mL ± 2.096). One sample (UC5) was removed from further analysis as no detectable DNA was extracted.

16S rRNA gene sequencing and taxonomic profiling was used to identify the origin of circulating BEVs in HC, CD, and UC plasma. Sequence data were analyzed using the Dadaist2 pipeline. Using MicrobiomeAnalyst, a total of 392,177 quality filtered amplicon sequence variants (ASVs) were generated, with 14,525 ± 11,270 average reads per sample.

**Figure 4:**
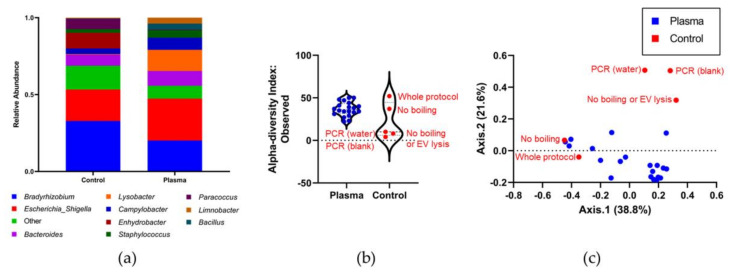
Taxonomic profiles between protocol control samples and plasma are distinct. (**a**) The relative abundance chart shows the top 10 most dominant taxa at the genus level. (**b**) Microbial richness measured by α-diversity in protocol controls was lower than that of plasma samples in three cases but comparable to plasma in two (ASV-level Observed Index: number of unique ASVs observed per sample). (**c**) The β-diversity PCoA plot shows that three protocol control samples (red) form a cluster that is significantly distinct from the plasma samples; however, two samples cluster alongside plasma (blue) (ASV-level; Bray–Curtis Index). Violin plot with individual values demonstrating frequency distribution: dotted lines represent the upper and lower quartiles, and the central dashed line represents the median.

Two samples were removed from the analysis due to low read counts (HC5 and CD8). The ASVs were mapped to 65 genera with the most dominant being *Bradyrhizobium, Escherichia/Shigella*, *Bacteroides,* and *Lysobacter* (Figure 4a). A full list of taxa (ASV) abundance is provided in Table S1.

To identify 16S rRNA gene amplicons originating from DNA contamination during sample processing, taxonomic profiles from protocol control samples and plasma were compared. With respect to α-diversity (microbial richness), three controls (no boiling or EV lysis, and PCR only (water), and PCR only blank) exhibited lower microbial richness than the plasma samples, whereas two controls (whole protocol and no boiling) were more comparable to plasma (ASV-level Observed Index: number of unique ASVs observed per sample; Figure 4b). β-diversity (microbial community composition dissimilarity) was visualized using a PCoA plot with Bray-Curtis Index and showed that the same three protocol control samples were significantly distinct from the plasma samples and two again clustered with the plasma samples (ASV-level; Figure 4c). 

The protocol control samples were designed to identify at which stage contamination is introduced, with one step of the protocol being removed for each control sample. The two protocol control samples that clustered alongside the plasma samples suggest that contamination is introduced after the EV lysis steps. This can be visualized as an increase in genera abundance (Appendix Figure A1a,b) and microbial richness (α-diversity index: Observed, ASV-level; Appendix Figure A1c) in the whole protocol and no boiling samples. Using CUA, significantly differential genera between plasma and the protocol controls were identified as *Lysobacter* (FDR *p*-value: 0.000013938, *Bacillus* and *Pseudomonas* (FDR *p*-values: 0.001606), and *Xanthobacter* (FDR *p*-value: 0.0020979), *Limnobacter* (FDR *p*-value: 0.0028168), *Acinetobacter* (FDR *p*-value: 0.005619), *Kocuria* (FDR *p*-value: 0.0076808), and *Brachybacterium* (FDR *p*-value: 0.015392) (Table S2). 

**Figure 5:**
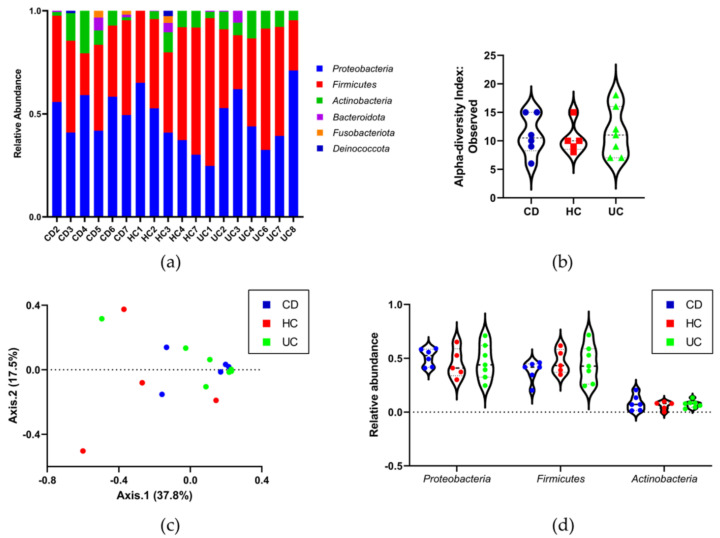
Microbial diversity and richness. Sequence-based filtering was performed to remove representative protocol control reads with >20% prevalence in the plasma samples. (**a**) The most abundant taxa at the phylum level were *Proteobacteria*, *Firmicutes,* and *Actinobacteria*. (**b**) Microbial richness and diversity were not significantly different between groups. The α-diversity plot shows no significant difference between groups (ASV-level Observed Index: number of unique ASVs observed per sample and one-way ANOVA *p*-value: 0.89198), and (**c**) the β-diversity PCoA plot also shows no significant differences between the groups (ASV level: Bray-Curtis Index and permutational MANOVA (PERMANOVA) *p*-value: 0.276). (**d**) There were also no significant changes in relative abundance at the phylum level (*Proteobacteria*, *Firmicutes,* and *Actinobacteria* one-way ANOVA *p*-values: HC vs. CD 0.7044, 0.4811, and 0.6117, respectively; HC vs. UC 0.9763, 0.9646, and 0.8331, respectively; FDR *p*-values: HC vs. CD 0.4954, 0.3127, and 0.4148, respectively; HC vs. UC 0.8620, 0.8311, and 0.6269, respectively. Violin plots with individual values demonstrating frequency distribution: dotted lines represent the upper and lower quartiles, and the central dashed line represents the median. Healthy controls (HC; red), Crohn’s disease (CD; blue), and ulcerative colitis (UC; green).

Sequence-based filtering was performed to remove representative protocol control reads with >20% prevalence in the plasma samples. Two further samples were removed from the analysis due to low read counts (HC6 and CD1). A total of 14,864 ASVs were identified from the filtered plasma-derived BEV samples, with 743 ± 586 average reads per sample (Table S3). At the phylum level, the most abundant taxa were *Proteobacteria*, *Firmicutes,* and *Actinobacteria* (Figure 5a). 

The α-diversity analysis showed no significant difference in microbial richness between groups (ASV-level Observed Index and one-way ANOVA *p*-value: 0.89198; Figure 5b). β-diversity analysis showed no significant differences in microbial community composition between the groups (ASV level PCoA plot with Bray–Curtis Index and permutational MANOVA (PERMANOVA) *p*-value: 0.276; Figure 5c). The overall microbial composition also did not reveal any differences at the phylum level (*Proteobacteria*, *Firmicutes,* and *Actinobacteria* one-way ANOVA *p*-values: HC vs. CD 0.7044, 0.4811, and 0.6117, respectively; HC vs. UC 0.9763, 0.9646, and 0.8331, respectively; FDR *p*-values: HC vs. CD 0.4954, 0.3127, and 0.4148, respectively; HC vs. UC 0.8620, 0.8311, and 0.6269, respectively; Figure 5d). 

**Figure 6:**
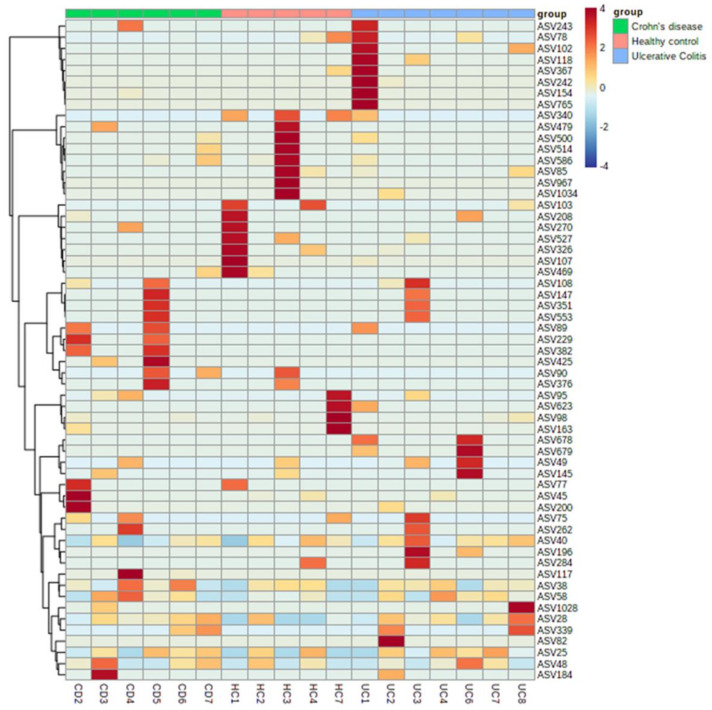
Hierarchical clustering and heatmap visualization at the feature (ASV) level. Heatmap clustering (Euclidean distance measure and Ward clustering algorithm) identified 59 ASVs observed in all groups (HC, Red; CD, green; and UC, blue). In HC, most observed taxa (dark red) were *Proteobacteria* including *Brevundimonas* genus and *Firmicutes* including *Bacillus, Enterococcus, Staphylococcus,* and *Streptococcus* genus. In CD and UC, most taxa were *Proteobacteria* including *Escherichia/Shigella*, *Paracoccus*, *Xanthobacter*, *Pseudomonas,* and *Lysobacter* genus as well as *Actinobacteria* including *Brachybacterium* and *Brevibacterium* genus, and *Firmicutes* including *Staphylococcus*, *Streptococcus, Lactococcus,* and *Lactobacillus* genus.

Heat map clustering at the ASV level revealed 59 specific ASVs observed in all groups (Figure 6). In HC, most highlighted taxa (red) were *Proteobacteria* including *Brevundimonas* genus and *Firmicutes* including *Bacillus, Enterococcus, Staphylococcus,* and *Streptococcus* genus. In CD and UC, they were *Proteobacteria* including *Escherichia/Shigella*, *Paracoccus*, *Xanthobacter*, *Pseudomonas,* and *Lysobacter* genus as well as *Actinobacteria* including *Brachybacterium* and *Brevibacterium* genus, and *Firmicutes* including *Staphylococcus*, *Streptococcus, Lactococcus,* and *Lactobacillus* genus. However, these taxa were each typically found to be elevated among only one member of each group and no differences between groups were found to be statistically significant using CUA. A full list of taxa is provided in Table S4.

## 4. Discussion

BEVs in body fluids such as plasma have not been as widely studied as mammalian EVs, likely due to the difficulties associated with their isolation [52]. We developed a rapid, cost-effective protocol to isolate BEV-associated DNA and used 16S rRNA gene sequencing to identify bacterial origins of the blood microbiome. To test the feasibility of using our BEV DNA isolation protocol to profile the blood microbiome of healthy and diseased individuals, we undertook a pilot study to compare the blood microbiome of healthy individuals and patients with CD and UC. To our knowledge the presence of BEVs and associated blood microbiome profile in patients with IBD has not been previously investigated. Using 16S rRNA gene sequencing, we found that the BEV-associated blood microbiota composition was not distinct between IBD, HC, or protocol control groups.

As an initial experiment prior to the main study, we compared two commonly used EV isolation methods: ultracentrifugation and SEC [53,54] as well as boiling, which is frequently used for plasma BEV-associated DNA isolation [26,27,55,56,57]. Based upon the relative amplification of the V3–V4 region of the 16S rRNA gene per mL of plasma, SEC and boiling were optimal for maximal DNA isolation (Figure 3a).

Although SEC using IZON qEV columns successfully separated EVs from protein contaminants (Appendix Figure A2), the boiling protocol was chosen as it had the lowest level of bacterial DNA contamination in the protocol control (water) sample (Figure 3a). The boiling protocol is also the least labor intensive and requires no specialist equipment such as SEC columns or an ultracentrifuge, which are important factors for potential use of the protocol as a high throughput, routine blood test in a clinical setting. In addition, TEM imaging confirmed that boiling reduced levels of contaminating extracellular protein aggregates (Figure 3c). It should be noted that if detailed functional, biochemical, or biophysical studies of highly purified BEVs are required, more stringent protocols such as that developed by Tulkens et al. using SEC and/or density-gradient centrifugation should be implemented [52,58,59]. 

Extracellular DNA can cause contamination issues in sequencing studies and is often ignored or overlooked in BEV-associated DNA sequencing protocols. To eliminate this potential problem, we pre-treated all plasma samples with DNase I prior to boiling and DNA isolation, the efficiency of which was confirmed by spiking plasma samples with DNA from a non-gut related bacterium, *B. subtilis* (Figure 3c). This data highlights the importance of the removal of extracellular DNA in low-biomass samples such as plasma prior to 16S rRNA gene amplification and sequencing. 

Another limitation of using 16S rRNA gene sequencing-based analysis of these low-biomass samples is the potential for contamination from laboratory reagents [60,61,62]. We found that bacterial DNA appears to be introduced after the EV lysis stage, likely from the SDS lysis buffer and DNA isolation kit reagents (Appendix Figure A1), rendering the protocol control samples indistinguishable from plasma samples in terms of α- and β-diversity. This contamination from DNA kits is often referred to as the ‘kit-ome’ and is a common problem in low-biomass sequencing studies [60,62] which can result in the microbiome between control and disease samples being indistinguishable [61,63,64,65], as found in this study. 

Therefore, our pilot study highlights that appropriate controls need to be included to identify sources of laboratory contamination and sequencing data needs to be interpreted with care. For this study, we aimed to minimise laboratory contamination by processing all plasma and protocol control samples as a single batch for EV isolation, PCR, and sequencing to produce robust and reliable results and avoid any batch-effect in processing and sequencing. Additionally, our 16S rRNA gene sequencing data analysis first involved identifying and removing the representative protocol control reads from the plasma reads. This enabled us to identify the ‘kit-ome’ as a potential source of microbial DNA. Our finding of water-borne bacterial genera such as *Pseudomonas*, *Stenotrophomonas*, *Xanthomonas*, *Ralstonia,* and *Bacillus,* as well as soil and plant associated bacteria such as *Spingobacteriaceae*, *Bradyrhizobiaceae*, *Methylbacterium,* and *Phyllobacreriaceae* in the protocol control samples, mirrors that of previous studies [61,64,65,66]. Therefore, future studies will need to eliminate these sources of contamination alongside appropriate protocol controls.

The co-isolation of host derived EVs, including exosomes containing host DNA, can also reduce the purity of BEV isolates. However, separating low-abundance BEVs from mammalian EVs, proteins, and lipid particles is challenging [67]. The most abundant particles in plasma are lipoproteins, with only <1% being identified as EVs by a lipid bilayer [50,68]. The majority of the identified plasma particles are therefore likely to be lipoproteins and soluble proteins. Additionally, the overlap between EV and BEV size (30–1000 nm for EVs and 20–250 nm for BEVs) [52,69,70] makes separation by size difficult. Using NTA, we found that prior to processing, plasma contained 2.8 × 10^12^ nanoparticles per mL with a mean particle size of 94 nm (Figure 2a). This size distribution could not be confirmed in non-processed plasma using TEM due to the presence of contaminating proteins which adhered to the EM grids (Appendix Figure A2b). However, imaging of a patient plasma sample purified by SEC revealed intact, spherical vesicles with the characteristic cup-shape (Figure 2b) morphology of BEVs [16,18,20,71].

Therefore, future studies should focus on both increasing the concentration of BEV-associated DNA from plasma samples by using a greater starting volume of plasma (>1 mL) and including additional BEV specific isolation/concentration steps such as density-gradient centrifugation, which can separate BEVs from mammalian EVs, and liposomes or antibody-mediated depletion (immunomagnetic separation), which can separate mammalian EVs from BEVs.

Although the 16S rRNA analysis of BEV DNA showed that there was no significant difference in the blood microbiota richness, diversity, or community composition between groups, the presence of BEV DNA originating from dominant gut bacteria such as *Bacteroides, Bifidobacterium, Campylobacter*, *Cutibacterium*, *Escherichia_Shigella,* and *Streptococcus* [72,73] in the plasma of both IBD patients and healthy individuals suggests luminal antigens and bacterial products can translocate across the epithelial barrier and access the circulatory system. As the findings described here in our pilot study were from patients with established but non-active disease, it is not unexpected that there were no significant differences in the origin of circulating BEVs between groups. In IBD patients with active disease and a compromised intestinal epithelial barrier, it would be predicted that a greater number of luminal BEVs translocating from the gut to the circulation would be identified that might mirror the gut microbiota [74]. 

Our rapid BEV DNA isolation protocol would facilitate and aid future studies examining larger cohorts of patients, including those pre-diagnosis, pre-symptomatic, and post-diagnosis experiencing active, symptomatic disease that are required to test this hypothesis further. Comparison of our BEV blood test with traditionally used inflammatory biomarkers would provide further evidence for the expediency of our novel technology. A longitudinal study following patients’ pre-diagnosis through to diagnosis and treatment would also be invaluable. 

## 5. Conclusions

We have developed a rapid, cost-effective protocol to isolate BEV-associated DNA and used 16S rRNA gene sequencing to identify bacterial origins of the blood microbiome. From our pilot study, using small sample numbers, it was not possible to detect significant differences in microbial composition of plasma derived BEVs from healthy individuals versus IBD patients. Our simple protocol provides the means of undertaking a larger study to test the hypothesis that the gut microbiota of IBD patients is reflected in plasma BEVs and determine if they may be of use as a diagnostic aid in IBD.

## Figures and Tables

**Table 1:** Patient characteristics. Clinical characteristics and demographics of St Mark’s Hospital blood donors and healthy volunteers [33].

Characteristic	HC ^1^	CD ^2^	UC ^3^
n	7	8	8
Male/female	5/2	3/5	5/3
Mean age at sampling	44 (28–57) *	49 (24–73) *	56 (41–71) *
Mean age at diagnosis		37 (19–61) *	40 (24–53) *
Symptoms at sampling:			
Diarrhea/loose stools		3	2
Abdominal pain/bloating/flatulence		0	1
Peri-anal pain/itch/disease		1	1
Anal fissure/bleed/proctitis		1	2
None		5	3
Medications at sampling:			
IBD ^4^ medications		6	6
Non-IBD medications		1	3
None		1	1

^1^ Healthy control (HC), ^2^ Crohn’s disease (CD), ^3^ ulcerative colitis (UC) and ^4^ inflammatory bowel disease (IBD). * Mean (range).

## Data Availability

The data for this study have been deposited in the European Nucleotide Archive (ENA) at EMBL-EBI under accession number PRJEB46867 (https://www.ebi.ac.uk/ena/browser/view/PRJEB46867, accessed on 6 October 2021).

## Supplementary Materials

The following are available online at https://www.mdpi.com/article/10.3390/genes12101636/s1, Table S1: Taxa (ASV level) abundance in protocol control and plasma samples, Table S2: Differential genera in protocol control and plasma samples identified by classical univariate analysis (CUA), Table S3: Taxa (ASV level) abundance in healthy control (HC), Crohn’s disease (CD), and ulcerative colitis (UC) plasma samples, Table S4: Differential genera in healthy control (HC), Crohn’s disease (CD), and ulcerative colitis (UC) plasma samples identified by classical univariate analysis (CUA).
